# Papulo-nodules cutanés révélant un lymphome anaplasique systémique à grandes cellules

**DOI:** 10.11604/pamj.2015.21.57.6898

**Published:** 2015-05-25

**Authors:** Inssaf Ramli, Badredine Hassam

**Affiliations:** 1 Service de Dermatologie, CHU Ibn Sina, Rabat - Instituts, rue Famfdal Cherkaoui, BP 6527,10000 Rabat, Maroc

**Keywords:** Lymphome anaplasique systémique, papulo-nodules, ALK+, Systemic anaplastic lymphoma, papulo-nodules, ALK+

## Image en medicine

Le lymphome anaplasique à grandes cellules (LAGC) systémique est une pathologie rare qui ne représente que 2 à 8% des lymphomes chez l'adulte. Sur le plan clinique, il est caractérisé par une atteinte essentiellement ganglionnaire. L'atteinte cutanée représente 10% de l'ensemble des atteintes extra-ganglionnaires. Ces manifestations cutanées peuvent être spécifiques liées à l'infiltration tumorale, ou non spécifiques dites paranéoplasiques. L'histologie et l'immunohistochimie sont deux examens capitaux pour le diagnostic positif, Les cellules néoplasiques des LAGC expriment dans tous les cas l'antigène CD30. 70% de ces lymphomes chez l'adulte sont associés à des translocations chromosomiques récurrentes impliquant la protéine ALK. La plupart des études ont indiqué l'existence de différences nettes entre les formes ALK+ et ALK– dont la plus importante est d'ordre pronostique. Le traitement du LAGC systémique comprend habituellement une polychimiothérapie type CHOP. D'autres traitements sont possible à savoir une radiothérapie, une greffe de cellules souches et parfois l'immunothérapie en couplant une immunotoxine à un anticorps anti-CD30. Nous rapportons le cas d'un patient de 52 ans ayant un LAGC systémique révélé par des papulo- nodules cutanés infiltrés à évolution ulcéreuse siégeant au niveau de la face interne de la cuisse gauche. L’étude immuno-histologique confirmait le diagnostic de LAGC. Un bilan d'extension révélait une localisation ganglionnaire et hépatique. Une polychimoithérapie type CHOP était indiquée cependant le patient est décédé, par un état de choc septique, avant de commencer la première cure de traitement.

**Figure 1:**
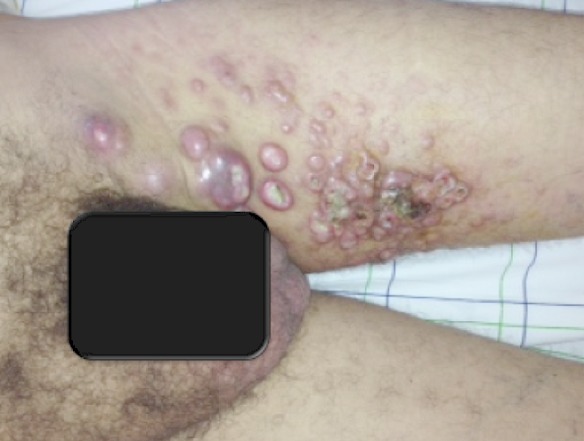
Papulo-nodules ulcérés de la cuisse

